# Interleukin-18 Gene Polymorphisms and Rheumatoid Arthritis Susceptibility: An Umbrella Review of Meta-Analyses

**DOI:** 10.1155/2024/6631033

**Published:** 2024-01-31

**Authors:** Yuehong Chen, Yali Ye, Huan Liu, Zhongling Luo, Qianwei Li, Qibing Xie

**Affiliations:** Department of Rheumatology and Immunology, West China Hospital, Sichuan University, Chengdu 610041, China

## Abstract

This study systematically analyzes the association between interleukin-18 (IL-18) gene polymorphisms and rheumatoid arthritis (RA) susceptibility. The electronic databases Ovid MEDLINE, Ovid Excerpta Medica Database, and Cochrane Library were searched to identify meta-analyses that included case–control studies reporting IL-18 gene polymorphisms and RA susceptibility. Data were reanalyzed using Review Manager Software 5.1, and Mantel–Haenszel random effects were applied for the five genetic models: allelic, recessive, dominant, homozygote, and heterozygote. The effect size of odds ratios (ORs) and their corresponding 95% confidence interval (CI) were calculated. A total of seven meta-analyses with poor quality were included. The IL-18 polymorphisms -607 A/C, -137 C/G, -920 T/C, and -105 C/A have been reported. With weak evidence, IL-18 -607 A/C polymorphisms were associated with a reduced risk of RA susceptibility using the allele model (OR = 0.76, 95% CI: 0.61 − 0.93, *p*=0.01), dominant model (OR = 0.67, 95% CI: 0.50 − 0.90, *p*=0.008), homozygote model (OR = 0.57, 95% CI: 0.35 − 0.91, *p*=0.02), and heterozygote model (OR = 0.71, 95% CI: 0.54 − 0.93, *p*=0.01) in the overall population. IL-18 gene polymorphisms and RA susceptibility are affected by ethnicity: With weak evidence, IL-18 -137 C/G polymorphisms were related to reduce RA susceptibility in the Asian population (allele model: OR = 0.59, 95% CI: 0.40 − 0.88, *p*=0.01; dominant model: OR = 0.57, 95% CI: 0.37 − 0.89, *p*=0.01; heterozygote model: OR = 0.60, 95% CI: 0.38 − 0.94, *p*=0.03). IL-18 -607 A/C gene polymorphisms are a protective factor for RA susceptibility in the overall population, and IL-18 −137 C/G gene polymorphisms are a protective factor for RA susceptibility in the Asian population. Further studies are needed to confirm these results owing to the limitations of the included studies.

## 1. Introduction

Rheumatoid arthritis (RA), a chronic autoimmune disease characterized by articular synovitis and the presence of autoantibodies, is a consequence of the complex interplay between inheritance, environmental triggers, and the immune system [1]. The etiology of RA remains poorly understood. However, available evidences suggest that an imbalance within the immune system plays a pivotal role [2]. The innate immunity acts as the primary defense against pathogenic bacteria, followed by the stimulation of adaptive immune response. This mechanism involves diverse cell populations and their secreted cytokines. These cells are categorized into three main subsets: CD4+ T helper 1 (Th1), Th2, and Th17 subsets, based on the transcription factors, cytokine expression, and cellular functions [2]. Cytokines, which are low molecular weight peptides, can be synthesized by different cell types, including various immune cell subpopulations, and they govern immune processes [3]. Proinflammatory cytokines, such as interleukin- (IL-)18, have been implicated in the promotion of autoimmune inflammation and tissue damage [4].

IL-18 belongs to the IL-1 superfamily and is a proinflammatory cytokine [5, 6]. IL-18, also known as interferon-*γ*- (IFN-*γ*-) inducing factor, can promote IFN-*γ* production [7–9]. It has proinflammatory activity and immunostimulatory effects, regulating both innate and adaptive immune responses. Thus, it plays an important role in the pathogenesis of a wide range of diseases, such as RA as well as systemic lupus erythematosus, type 1 diabetes, psoriasis, inflammatory bowel diseases, metabolic syndrome, cardiovascular diseases, lung inflammatory diseases, and cancer. Therefore, IL-18 has been a potential treatment target. IL-18 is secreted by macrophages in response to stimuli such as viruses and bacteria and has been implicated in the pathogenesis of RA. In RA, posttranslational processing by caspase-1 contributes to the activation and maturation of IL-18, after which IL-18 mediates downstream signaling and is involved in regulating inflammation during the onset and maintenance processes [10]. IL-18 can promote T helper 1 (TH1) lymphocytes and macrophages to produce IFN-*γ*, increase natural killer cell cytotoxicity, and indirectly stimulate osteoclast formation in RA synovitis [11]. IL-18 is expressed in the synovial tissue of patients with RA and is associated with joint inflammation severity, acute phase response, and the gene expression of IL-1*β* and tumor necrosis factor alpha (TNF*α*) [3]. Serum and synovial fluid levels of IL-18 in patients with RA were found to be higher than those in healthy controls or patients with osteoarthritis [12–15].

A previous study reported that the occurrence of RA was related to inheritance, and the possibility of inheritance was as high as 60% [16]. In addition, both human leukocyte antigen (HLA) and non-HLA gene polymorphisms contribute to RA susceptibility, and more than 120 predisposed gene loci have been reported for RA [17, 18]. Some of the gene polymorphisms are population specific. For example, the +49 A/G (rs231775) polymorphism in cytotoxic T lymphocyte-associated antigen-4 (CTLA-4) is associated with RA susceptibility in Asians, and the -174 G/C (rs1800795) polymorphism in IL-6 is correlated with RA susceptibility in Europeans [19]. The relationships between polymorphisms in several gene sites of IL-18, such as -607 A/C, -137 C/G, -920 T/C, -105 C/A, and susceptibility to RA have been assessed; however, no consistent results have been reported [20–26]. Therefore, we performed this umbrella review of meta-analyses using up-to-date evidence to systematically review the association between IL-18 polymorphisms and RA susceptibility.

## 2. Methods

### 2.1. Study Design

Using an umbrella review, this study systematically assessed the association between IL-18 polymorphisms and RA susceptibility.

### 2.2. Inclusion and Exclusion Criteria

We included the meta-analysis of case–control studies. The case group comprised participants who were diagnosed with RA, whereas the control group comprised healthy participants. IL-18 polymorphisms were detected by polymerase chain reaction or Taqman methods, and the numbers of each genotype were reported. There were no limitations to the gene sites of IL-18. A study was excluded if it was a narrative review, systematic review without using systematic methods recommended by the Cochrane handbook, duplicate publication, study protocol, or executive summary.

### 2.3. Search Strategy

The electronic databases Ovid MEDLINE, Ovid Excerpta Medica Database (EMBASE), and Cochrane Library were searched using a combination of relevant MeSH terms and keywords on April 1, 2022. The search terms used were “systematic review,” “meta-analysis,” “rheumatoid arthritis,” and “interleukin-18.” The reference lists of the included studies and excluded reviews were manually checked to identify possible eligible studies. The detailed search strategy is reported in the *Supplementary 1*.

### 2.4. Study Selection

The retrieved studies were exported to EndNote software to remove duplicates, and the remaining studies were exported to Microsoft Office Access to manage the study selection. A pair of reviewers screened the potential eligible studies through titles, abstracts, and full texts on the basis of the inclusion and exclusion criteria. Disagreements were resolved through discussion or by a third reviewer. The study selection process was performed on the basis of preferred reporting items for systematic reviews and meta-analyses (PRISMA) flow diagram.

### 2.5. Data Extraction

A pair of authors collected the following information from the included meta-analyses: family name of first author, year of publication, country of first author, study number, number of participants in a meta-analysis, number of each genotype, study search date, searched databases, reported gene sites in IL-18, analyzed gene model, methods and results of the publication bias assessment, conclusion, and ethnicity. Disagreements were resolved through discussion or by a third reviewer. Microsoft Office Access 2013 was used to manage the data extraction. We extracted the number of each genotype from a meta-analysis rather than the original case–control studies included in the meta-analysis.

### 2.6. Quality Assessment of the Included Meta-Analyses and Evidence Grading

The Overview Quality Assessment Questionnaire was applied to assess the quality of the included meta-analysis. It had a total of nine items, and for each item, the following applied: if the question was correctly performed, the answer was yes; if the study did not describe whether the question was performed, the answer was unclear; and if the question was not performed or wrongly performed, the answer was no. The following criteria were used to estimate the overall quality of a study: if the answer was yes for all items, the quality was high; if one or more unclear answers were presented, the quality was moderate; if there was no answer for the second, fourth, sixth, or eighth item, the quality was low; and if more than one answer was presented for the second, fourth, sixth, or eighth item, the quality was critically low [27]. Two reviewers performed the quality assessment. Disagreements were resolved through discussion or by a third reviewer.

The GRADE (grading of recommendations, assessment, development, and evaluation) working group classification was used to classify the evidence, which included the following: convincing, highly suggestive, suggestive, weak, and nonsignificant. The evidence was graded using the sample size, heterogeneity, publication bias, and effect magnitude [28, 29].

### 2.7. Data Process

Data were analyzed by Review Manager Software 5.1 (Cochrane Collaborate, Oxford, UK). To repool the data from the individual case–control studies that were included in the meta-analyses, we used Mantel–Haenszel statistical method for the five comparison models: allelic (a (minor allele) vs. A (wild allele)), recessive (aa vs. Aa + AA), dominant (aa + Aa vs. AA), homozygote (aa vs. AA), and heterozygote (Aa vs. AA). All data were pooled using random effects model. The effect size of odds ratios (ORs) and their corresponding 95% confidence interval (CI) were calculated. Heterogeneity between studies was assessed by using Cochrane's Q-statistic and was quantified by *I*^2^ value with *p*=0.1. The data would be pooled if more than two studies reported the same outcome. Publication bias was assessed using a funnel plot by visual inspection if at least 10 studies were included on the basis of Cochrane handbook. In addition, the Eggers test was performed to quantify the *p* value for publication bias.

## 3. Results

### 3.1. Characteristics of Included Studies

**Figure 1:**
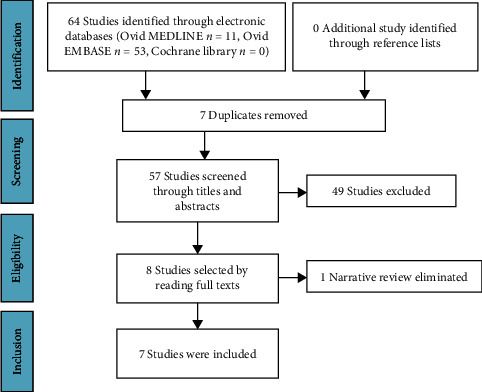
PRISMA study selection flowchart.

A total of 64 studies were retrieved from the electronic databases of Ovid MEDLINE (*n* = 11), Ovid EMBASE (*n* = 53), and Cochrane Library (*n* = 0). After excluding duplicates, title and abstract screening, and full-text screening, seven studies [20–26] remained (Figure 1). No additional studies were identified after checking the reference list. Of the seven included studies, five were performed in China, and two in Korea. All included studies were published between 2011 and 2016. The number of included studies ranged from 1 to 14, with 201–3,728 RA cases and 218–3,213 controls in the systematic review and meta-analysis. IL-18 gene polymorphisms -607 A/C, -137 C/G, -920 T/C, and-105 C/A were reported by the included meta-analyses that assessed the association between IL-18 gene polymorphisms and RA susceptibility (Table 1).

### 3.2. IL-18 Gene Polymorphisms and RA Risk in the Overall Population

A total of 12 studies were included for the reanalysis. With weak evidence, the results suggested that IL-18 -607 A/C gene polymorphisms were associated with a reduced risk of RA susceptibility using the allele model (OR = 0.76, 95% CI: 0.61 − 0.93,*p*=0.01), dominant model (OR = 0.67, 95% CI: 0.50 − 0.90, *p*=0.008), homozygote model (OR = 0.57, 95% CI: 0.35 − 0.91, *p*=0.02), and heterozygote model (OR = 0.71, 95% CI: 0.54 − 0.93, *p*=0.01) (*Supplementary 2*, Table 2). Totally, nine studies were included to assess the correlation between IL-18 -137 C/G gene polymorphisms and RA susceptibility. IL-18 -137 C/G gene polymorphisms were not associated with RA susceptibility using any genetic model (*Supplementary 2*, Table 2). As for IL-18 -920 T/C, only two studies reported the relevant data. They suggested with weak evidence that IL-18 -920 T/C was associated with reduced RA susceptibility using the allele model (OR = 0.85, 95% CI: 0.76 − 0.95, *p*=0.004), while the other genetic models did not suggest a correlation (Table 2).

### 3.3. IL-18 Gene Polymorphisms and RA Susceptibility by Ethnicity Subgroup Analysis

Five studies in total were included to reanalyze the association between IL-18 -607 A/C polymorphisms and RA susceptibility by ethnicity (Caucasian or Asian), and the results suggested that a reduced risk of RA susceptibility was not found in Caucasian or Asian populations by using any of the genetic analysis models (Table 2). As for the association between IL-18 -137 C/G polymorphisms and RA susceptibility, we analyzed five and two studies for Caucasian and Asian populations, respectively. A reduction in RA susceptibility with weak evidence was only found in the Asian population when using the allele model (OR = 0.59, 95% CI: 0.40 − 0.88, *p*=0.01), dominant model (OR = 0.57, 95% CI: 0.37 − 0.89, *p*=0.01), and heterozygote model (OR = 0.60, 95% CI: 0.38 − 0.94, *p*=0.03) (Table 2). No data for ethnicity subgroup analysis were available for the correlation between IL-18 -920 T/C polymorphisms and RA susceptibility.

### 3.4. Quality of Included Reviews

None of the systematic reviews and meta-analyses reported whether the study selection was performed by a pair of reviewers, and none performed a quality assessment of the included case–control studies as well. Overall, the quality of the included reviews was poor (Table 3).

### 3.5. Publication Bias

**Figure 2:**
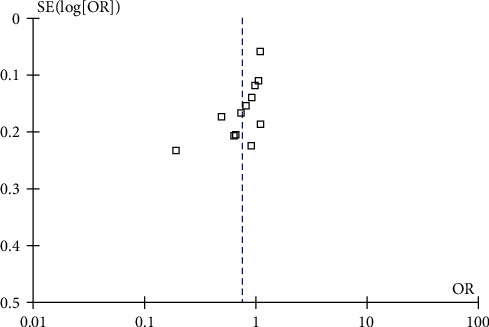
The funnel plot of the association between IL-18 -607 A/C gene polymorphisms and RA susceptibility in the overall population according to the allelic model.

A funnel plot showing the publication bias was presented if there were more than 10 studies included based on Cochrane handbook. The association between IL-18 -607 A/C gene polymorphisms and RA susceptibility was reported by 12 studies in the allele model, dominant model, recessive model, homozygote model, and heterozygote model in the overall population. We reported the representative funnel plot of the association between IL-18 -607 A/C gene polymorphisms and RA susceptibility in the overall population according to the allelic model. By visual inspection, the unsymmetry of the funnel plot suggested that a publication bias was possible (Figure 2). The Eggers test reported that the *p* value was 0.011.

## 4. Discussion

Our study found that IL-18 -607 A/C gene polymorphisms are a protective factor for RA susceptibility in the overall population, which is consistent with the results reported by Yuan et al. [30]. Yuan et al. [30] genetically analyzed data from two datasets, namely the GWAS data of Okada et al. [31] and the FinnGen consortium (R5) [32], using a Mendelian randomization design. They found that IL-18 levels were possibly associated with RA risk (OR = 1.07, 95% CI: 1.00 − 1.15) and that IL-18 levels appeared to be the genetic predisposition to seronegative RA risk (OR = 1.18, 95% CI: 1.02 − 1.36) [30].

In addition to RA predisposition, increasing evidence on the association between IL-18 polymorphisms and other disease conditions has also been reported by systematic reviews and meta-analyses. The -607 C/A and/or -137 G/C polymorphisms in IL-18 may confer susceptibility to tuberculosis, especially in Asians [33]; inflammatory bowel disease, including both Crohn's disease and ulcerative colitis in Asians and Africans [34–36]; vasculitis [37] and systemic lupus erythematosus in Europeans and Asians [38]; Behcet's disease [39], various types of liver diseases [40, 41], hepatitis B virus in East Asians and hepatitis C virus infection in South Asians [42] and Asian populations [43]; diabetic nephropathy in Asians [44]; allergic asthma and allergic dermatitis [45–47], Alzheimer's disease in Asians [48, 49]; periodontitis [50], coronary artery disease in East Asians [51, 52]; head and neck cancer risk, especially nasopharyngeal cancer, in Asian populations [53]; breast cancer in East Asians from China [54, 55]; and prostate cancer in Asian and Caucasian populations [56].

IL-18 has been reported to be a potential clinical biomarker. It can be used to differentiate macrophage activation syndrome (MAS) from other subtypes of hemophagocytic lymphohistiocytosis as MAS exhibits high levels of IL-18 (>10,000 pg/mL). It can also be used to predict the development of MAS and systemic juvenile idiopathic arthritis [57, 58]. Thus, IL-18 is a potential target for the treatment of MAS [9]. In addition, IL-18 can be used as a biomarker to distinguish active adult-onset Still's disease (AOSD) from COVID-19; IL-18 is 68-fold higher in patients with active AOSD than in those with severe COVID-19. Moreover, IL-18 is an important predictor of active AOSD and has a diagnostic value for the disease with a sensitivity and specificity of 91.3% and 95.8%, respectively, when the cutoff value is 190.5 pg/mL [59]. Furthermore, IL-18 can be used as a biomarker for acute kidney injury, as IL-18 might be involved in the development and progression of acute kidney injury, and patients with high urine IL-18 levels benefit from anti-IL-18 treatment [60]. In diabetic nephropathy, serum IL-18 levels are elevated and can be a predictive biomarker for the initiation of diabetic nephropathy in patients with diabetes. Both urinary and serum IL-18 levels are positively associated with the progression of renal injury and renal dysfunction at an early stage [61].

The receptor of IL-18 is a heterodimer consisting of IL-18R*α* and IL-18R*β*. After binding to its receptor, IL-18 mainly regulates two signaling pathways: one through IL-1R-associated kinases 1–4 to stimulate nuclear factor kappa B and another through TNF-associated factor 6 to stimulate mitogen-activated protein kinase, both eventually contributing to gene expression [8]. The biological actions of IL-18 are pleiotropic, which are dependent on the cytokine and immune cell milieu [8]. In the presence of IL-12, IL-18 can promote TH1 cells to secrete IFN-*γ* and T cells to produce IFN-*γ*, TNF-*α*, and Fas ligand to combat intracellular microbes, and in combination with IL-2, IL-18 induces natural killer T cells to release IL-4, IL-9, IL-13, and CD40 ligand to eliminate extracellular microbes. In the presence of IL-3, IL-18 can stimulate mast cells to produce IL-4, IL-13, and chemical mediators to control intestinal nematodes. IL-18 is also involved in regulating metabolism and is beneficial for metabolic hemostasis as it inhibits appetite and accelerates energy expenditure. Furthermore, as a potent cytokine, IL-18 can induce the production of other proinflammatory cytokines, transcription factors, and adhesion molecules, such as TNF-*α*, IL-1*β*, TGF-*β*, intercellular adhesion molecule 1, E-selectin, and vascular cell adhesion molecule-1, and the activation of IFN-*γ* [61].

Although our study included all the available evidence involving the reported gene sites of IL-18 polymorphisms associated with RA, there are several limitations regarding the quality of the meta-analyses included in this study. First, the quality of included meta-analyses was poor as none of them reported whether the study selection was performed by a pair of reviewers. In addition, they did not perform a quality assessment of the included case–control studies, which may compromise the credibility of the results. Second, the sample sizes of the studies were relatively small, the heterogeneity was obvious in several outcomes, and the effect magnitude was small. Third, publication bias might be existed. All this contributed to our grading of “weak evidence,” and the results should be explained by caution.

Even though currently it is only possible to identify weak evidence regarding the association between IL-8 and RA, it is still a worthwhile effort to reveal these possible correlations. Our analysis suggests that IL-18 -607 A/C gene polymorphisms are a protective factor for RA susceptibility in the overall population, and IL-18 -137 C/G polymorphisms are so for the Asian population. In summary, IL-18 might play a role in the pathogenesis of RA. Therefore, future studies on the pathogenesis mechanism of IL-18 and the targeting of IL-18 for the management of RA can lead to new therapeutic avenues.

## Figures and Tables

**Table 1:** Characteristics of included systematic reviews and meta-analyses.

Study	Country	Included study no.	Study search date	Included patients no.	Searched databases	Gene site	Analyzed gene model	Publication bias assessment	Conclusion
Cai et al. [20]	China	10 Studies from 8 articles for -607 A/C; 9 studies from 6 articles for -137 C/G	January 2013	2,662 Cases and 2,168 controls for -607 A/C; 1,331 cases and 1,468 controls for -137 C/G	PubMed, EMBASE, Spring-link, Web of Science, Wanfang (Chinese), and CNKI	-607 A/C, -137 C/G	Allele model, codominant model, dominant model, recessive model	Begg's funnel plot and Egger's test, no publication bias	IL-18 (-607 A/C) polymorphism may be associated with RA risk

Chen et al. [21]	China	7 Studies for -607 A/C; 5 studies for -137 C/G	April 2012	1,958 Cases and 1,707 controls for -607 A/C; 333 cases and 541 controls for -137 C/G	PubMed, EMBASE, Web of Science, CNKI, CBMD, and Wanfang (Chinese)	-607 A/C, -137 C/G	Allele model, dominant model, recessive model	Modified Egger's linear regression test, no publication bias	IL-18 (-607 A/C) polymorphism may confer susceptibility to RA in Chinese population, but not all Asians

Ji and Lee [22]	South Korea	7 Studies for -607 A/C; 5 studies for -137 C/G; 2 studies for -920 T/C	October 2012	2,944 Cases and 2,377 controls for -607 A/C; 1,319 cases and 1,211 controls for -137 C/G; 1,873 cases and 1,092 controls for -920 T/C	MEDLINE, PubMed	-607 A/C, -137 C/G, -920 T/C	Allele model, dominant model, recessive model, homozygote model	Begg's funnel plots and Egger's test, with publication bias for -607 A/C in all comparison models, without publication bias for -137 C/G in all comparison models	No significant association was found between two IL-18 (-607 A/C, -137 C/G) polymorphism and RA susceptibility in all subjects; IL-18 (-920 T/C) polymorphism is associated with RA susceptibility

Li et al. [23]	China	14 Studies from 13 articles for -607 A/C; 11 studies from 10 articles for -137 C/G; 2 studies for -920 T/C; 1 study for -105 C/A	May 1, 2015	3,728 Cases and 3,213 controls for -607 A/C; 1,982 cases 1,947 controls for -137 C/G; 1,873 cases 1,091 controls for -920 T/C; 201 cases 218 controls for -105 C/A	PubMed, Ovid, Cochrane Library, EMBASE, and China Knowledge Resource Integrated Database	-607 A/C, -137 C/G, -920 T/C, -105 C/A	Homozygote model, heterozygote model, dominant model, recessive model, additive model	Funnel plots and the Begg test, no publication bias	The -607 A/C, -920 T/C, and -105 A/C polymorphisms in IL-18 were significantly associated with increased RA risk. However, the -137 C/G polymorphism was not associated with RA risk under any genetic model

Pan et al. [24]	China	4 Studies from 3 articles	May 2010	756 Cases and 756 controls	MEDLINE and PubMed	-607 A/C	Recessive model, dominant model, allele model	Begg's funnel plot and Egger's test, no publication bias	Not enough evidence to indicate the association of IL-18 gene promoter -607 A/C polymorphism and the development of RA

Song et al. [25]	Korea	7 Studies for -607 A/C; 7 studies from 6 articles for -137 C/G	January 2013	2,894 Cases and 2,361 controls for -607 C/A; 1,596 cases and 1,478 controls for -137 C/G	MEDLINE and EMBASE	-607 A/C, -137 C/G	Allelic model, recessive model, dominant model, homozygote model	Funnel plots, no publication bias	IL-18 (-607 A/C and -137 C/G) polymorphisms are not associated with RA

Wen et al. [26]	China	5 Studies	Not reported	1,224 Cases and 1,368 controls	PubMed, EMBASE, and Cochrane library databases	-137 C/G	Dominant model, recessive model, additive model	Funnel plot and Egger's regression test, no publication bias	IL-18 (-137 C/G) was a risk factor for RA, especially for RA in Europeans

**Table 2:** Reanalysis results of association between interleukin-18 gene polymorphisms and rheumatoid arthritis.

Gene sites	Subgroup	Study number	Number of case/control	Heterogeneity (*I*^2^, *p*)	OR (95% CI), *p*	Grading
IL-18 -607 C/A						

Allele	Overall	12	6,838/5,816	86, <0.00001	**0.76 (0.61, 0.93), 0.01 ^*∗*^**	**Weak**
	Caucasian	5	2,506/1,900	91, <0.00001	0.72 (0.46, 1.12)	Nonsignificant
	Asian	5	3,926/3,068	86, <0.0001	0.73 (0.52, 1.02)	Nonsignificant

Recessive	Overall	12	3,419/2,908	80, <0.00001	0.73 (0.50, 1.06)	Nonsignificant
	Caucasian	5	1,253/950	86, <0.0001	0.73 (0.36, 1.50)	Nonsignificant
	Asian	5	1,963/1,534	79, 0.0006	0.68 (0.37, 1.24)	Nonsignificant

Dominant	Overall	12	3,419/2,908	81, <0.00001	**0.67 (0.50, 0.90), 0.008**	**Weak**
	Caucasian	5	1,253/950	86, <0.00001	0.63 (0.37, 1.07)	Nonsignificant
	Asian	5	1,963/1,534	86, <0.0001	0.64 (0.38, 1.07)	Nonsignificant

Hemozygote	Overall	12	1,744/1,389	84, <0.00001	**0.57 (0.35, 0.91), 0.02**	**Weak**
	Caucasian	5	657/445	89, <0.00001	0.55 (0.23, 1.34)	Nonsignificant
	Asian	5	1,003/781	86, <0.00001	0.53 (0.24, 1.14)	Nonsignificant

Heterozygote	Overall	12	2,658/2,271	77, <0.00001	**0.71 (0.54, 0.93), 0.01**	**Weak**
	Caucasian	5	1,070/799	78, 0.001	0.68 (0.43, 1.06)	Nonsignificant
	Asian	5	1,408/1,104	81, <0.0001	0.67 (0.40, 1.14)	Non-significant

IL-18 -137 G/C						

Allele	Overall	9	3,346/3,284	88, <0.00001	0.74 (0.52, 1.05)	Nonsignificant
	Caucasian	5	2,486/1,900	93, <0.00001	0.68 (0.39, 1.17)	Nonsignificant
	Asian	2	454/536	0, 0.58	**0.59 (0.40, 0.88), 0.01**	**Weak**

Recessive	Overall	9	1,673/1,642	83, <0.00001	0.70 (0.33, 1.51)	Nonsignificant
	Caucasian	5	1,243/950	91, <0.00001	0.57 (0.20, 1.61)	Nonsignificant
	Asian	2	227/268	0, 0.33	0.48 (0.11, 2.07)	Nonsignificant

Dominant	Overall	9	1,673/1,642	74, 0.0002	0.76 (0.56, 1.04)	Nonsignificant
	Caucasian	5	1,243/950	85, <0.0001	0.73 (0.46, 1.18)	Nonsignificant
	Asian	2	227/268	0, 0.82	**0.57 (0.37, 0.89), 0.01**	**Weak**

Hemozygote	Overall	9	1,089/1,085	83, <0.00001	0.67 (0.30, 1.47)	Nonsignificant
	Caucasian	5	765/593	91, <0.00001	0.54 (0.18, 1.59)	Nonsignificant
	Asian	2	190/205	0, 0.35	0.43 (0.10, 1.86)	Nonsignificant

Heterozygote	Overall	9	1,553/1,497	8, 0.37	0.89 (0.76, 1.06)	Nonsignificant
	Caucasian	5	1,136/824	23, 0.27	0.94 (0.75, 1.17)	Nonsignificant
	Asian	2	224/260	0, 0.95	**0.60 (0.38, 0.94), 0.03**	**Weak**

IL-18 -920 C/T						

Allele	Overall	2	3,746/2,182	0, 1.00	**0.85 (0.76, 0.95), 0.004**	**Weak**
Recessive	Overall	2	1,873/1,091	67, 0.08	0.29 (0.03, 3.36)	Nonsignificant
Dominant	Overall	2	1,873/1,091	0, 0.85	0.90 (0.76, 1.08)	Nonsignificant
Hemozygote	Overall	2	1,038/615	66, 0.08	0.30 (0.03, 3.35)	Nonsignificant
Heterozygote	Overall	2	1,572/837	0, 0.84	1.01 (0.84, 1.21)	Nonsignificant

^*∗*^Bold ORs with 95% CI indicate statistical meaning with *p* value less than 0.05.

**Table 3:** Study quality assessment results.

Study	(1) Were the search methods reported	(2) Was the search comprehensive	(3) Were the inclusion criteria reported	(4) Was selection bias avoided	(5) Were the validity criteria reported	(6) Was validity assessed appropriately	(7) Were the methods used to combine studies reported	(8) Were the findings combined appropriately	(9) Were the conclusions supported by the reported data	(10) What was the overall scientific quality of the overview
Cai et al. [20]	Yes	Yes	Yes	Unclear	Yes	Not performed	Yes	Yes	Yes	Low
Chen et al. [21]	Yes	Yes	Yes	Unclear	Unclear	Not performed	Yes	Yes	Yes	Low
Ji and Lee [22]	Yes	Yes	Yes	Unclear	Unclear	Not performed	Yes	Yes	Yes	Low
Li et al. [23]	Yes	Yes	Yes	Unclear	Yes	Not performed	Yes	Yes	Yes	Low
Pan et al. [24]	Yes	Yes	Yes	Unclear	Unclear	Not performed	Yes	Yes	Yes	Low
Song et al. [25]	Yes	Yes	Yes	Unclear	Yes	Not performed	Yes	Yes	Yes	Low
Wen et al. [26]	Yes	Yes	Yes	Unclear	Unclear	Not performed	Yes	Yes	Yes	Low

## Data Availability

All data are presented in tables or supplementary materials.

## Supplementary Materials

Supplementary 1 Detailed search strategy.

Supplementary 2 Supplemental figures from Figures S1 to S10.
