# Supplementary material for: Interleukin-18 Gene Polymorphisms and Rheumatoid Arthritis Susceptibility: An Umbrella Review of Meta-Analyses
Source: J Immunol Res. 2024 Jan 31;2024:6631033. doi: 10.1155/2024/6631033 (PMC10849815; doi:10.1155/2024/6631033)
Supplement: Supplementary 1 — Detailed search strategy. [file 6631033.f1.docx]

**Detailed Search Strategy**

**Search date April 1, 2022**

**Ovid MEDLINE(R) 1946 to March Week 4 2022 (n=11)**

**OVID Embase 1974 to 2022 March 31 (n=53)**

1 exp "systematic review"/

2 ("systematic literature review?" or "systematic review?" or "systematic overview?"). ti,ab.

3 exp "meta analysis"/

4 (meta?anal* or meta anal* or meta-anal* or metaanal* or metanal*).ti,ab.

5 ("methodologic* literature review?" or "methodologic review*" or "methodologic* overview?") .ti,ab.

6 ("quantitative review?" or "research integration?" or "research overview?") .ti,ab.

7 ("quantitative syntheses" or "quantitative synthesis").ti,ab.

8 ("integrative review?" or "integrative overview?" or "collaborative review?" or

"collaborative overview?") .ti,ab.

9 ("pooled analyses" or "pooled analysis").ti,ab.

10 ( "data extraction" or "data abstraction").ti,ab.

11 ("data synthesis" or "data syntheses").ti,ab.

12 1 or 2 or 3 or 4 or 5 or 6 or 7 or 8 or 9 or 10 or 11

13 rheumatoid arthritis.mp. or exp rheumatoid arthritis/

14 ((rheumatoid or reumatoid or revmatoid or rheumatic or reumatic or revmatic or

rheumat$ or reumat$ or revmarthrit$) adj3 (arthrit$ or artrit$ or diseas$ or condition$ or nodule$)).tw.

15 (felty$ adj2 syndrome).tw.

16 (caplan$ adj2 syndrome).tw.

17 (sjogren$ adj2 syndrome).tw.

18 (sicca adj2 syndrome).tw.

19 still$ disease.tw.

20 bechterew$ disease.tw.

21 13 or 14 or 15 or 16 or 17 or 18 or 19 or 20

22 IL-18.mp. or exp interleukin 18/

23 12 and 21 and 22

**Cochrane Library (n=0)**

1 “MeSH descriptor: [Arthritis, Rheumatoid] explode all trees” OR “(rheumatoid arthritis):ti,ab,kw” OR “(felty$ adj2 syndrome):ti,ab,kw” OR “(caplan$ adj2 syndrome):ti,ab,kw” OR “(sjogren$ adj2 syndrome):ti,ab,kw” OR “(sicca adj2 syndrome):ti,ab,kw” OR “still$ disease:ti,ab,kw” OR “bechterew$ disease:ti,ab,kw”

2 “interleukin-18:ti,ab,kw” OR “IL-18:ti,ab,kw” OR “IFN-gamma-inducing factor: ti,ab,kw”

3 #1 AND #2
