# Supplementary material for: Interleukin-18 Gene Polymorphisms and Rheumatoid Arthritis Susceptibility: An Umbrella Review of Meta-Analyses
Source: J Immunol Res. 2024 Jan 31;2024:6631033. doi: 10.1155/2024/6631033 (PMC10849815; doi:10.1155/2024/6631033)
Supplement: Supplementary 2 — Supplemental figures from Figures S1 to S10. [file 6631033.f2.docx]

Interleukin-18 Gene Polymorphisms and Rheumatoid Arthritis Susceptibility: An Umbrella Review of Meta-analyses

**Yuehong Chen†, Yali Ye†, Huan Liu, Zhongling Luo, Qianwei Li, Qibing Xie***

Department of Rheumatology and Immunology, West China Hospital, Sichuan University, Chengdu 610041, China

†These authors contributed equally to this work and share first authorship.

*Co-corresponding author, email: xieqibing1971@163.com, Department of Rheumatology and Immunology, West China Hospital, Sichuan University, 37 Guoxue lane, Chengdu 610041, China, Tel/Fax: +86-28-8542 2393

Supplemental figures: sFig1-sFig10


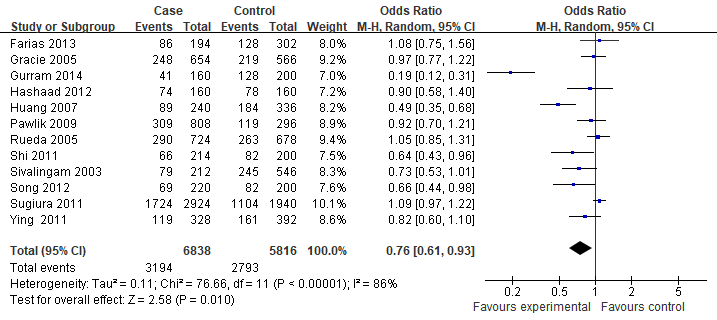


sFig.1 Forest plot of association between IL-18 -607A/C polymorphisms and RA susceptibility in allele model for the overall population


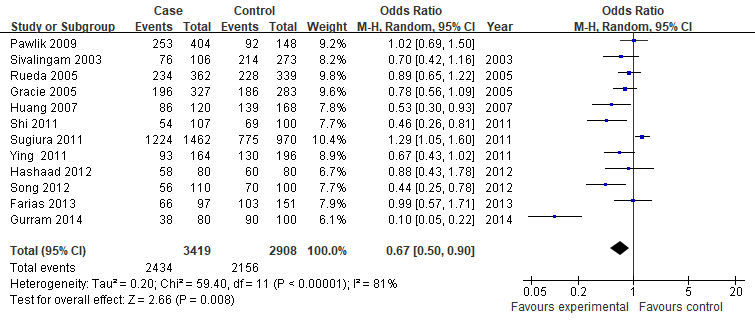


sFig.2 Forest plot of association between IL-18 -607A/C polymorphisms and RA susceptibility in dominant model for the overall population


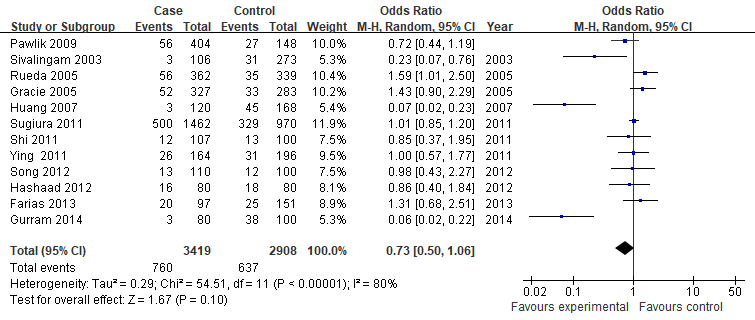


sFig.3 Forest plot of association between IL-18 -607A/C polymorphisms and RA susceptibility in recessive model for the overall population


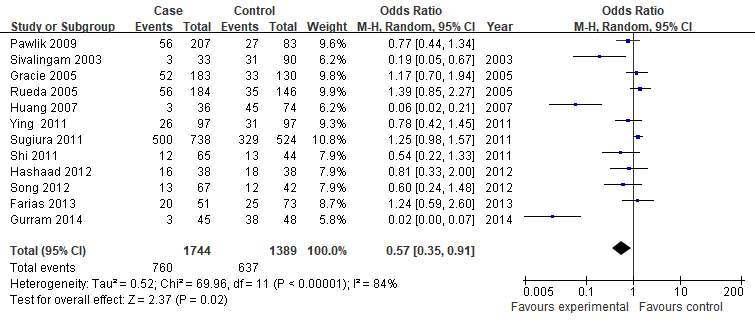


sFig.4 Forest plot of association between IL-18 -607A/C polymorphisms and RA susceptibility in hemozygote model for the overall population


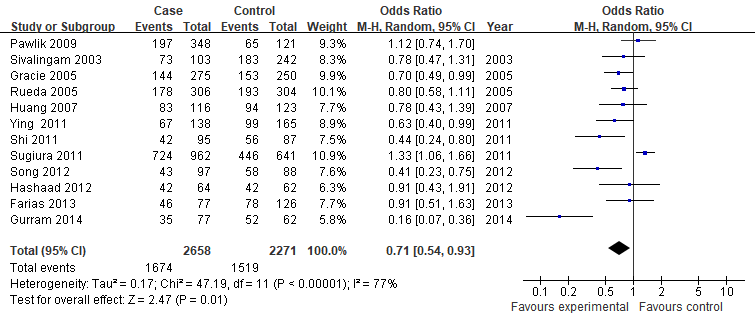


sFig.5 Forest plot of association between IL-18 -607A/C polymorphisms and RA susceptibility in heterozygote model for the overall population


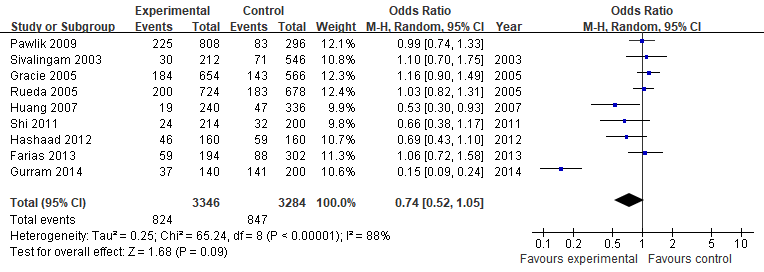


sFig.6 Forest plot of association between IL-18 -137C/G polymorphisms and RA susceptibility in allele model for the overall population


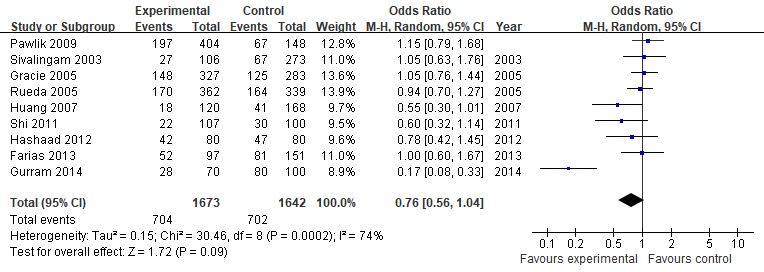


sFig.7 Forest plot of association between IL-18 -137C/G polymorphisms and RA susceptibility in dominant model for the overall population


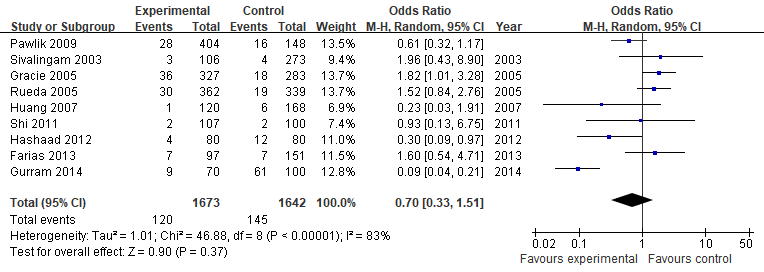


sFig.8 Forest plot of association between IL-18 -137C/G polymorphisms and RA susceptibility in recessive model for the overall population


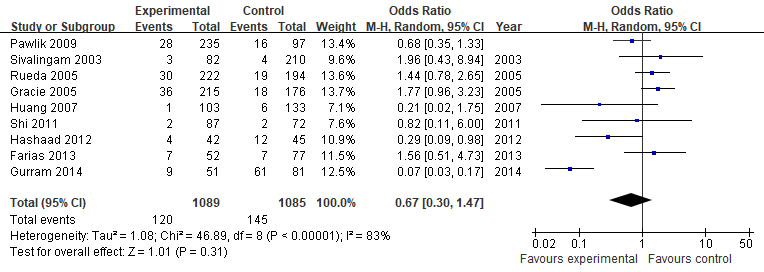


sFig.9 Forest plot of association between IL-18 -137C/G polymorphisms and RA susceptibility in hemozygote model for the overall population


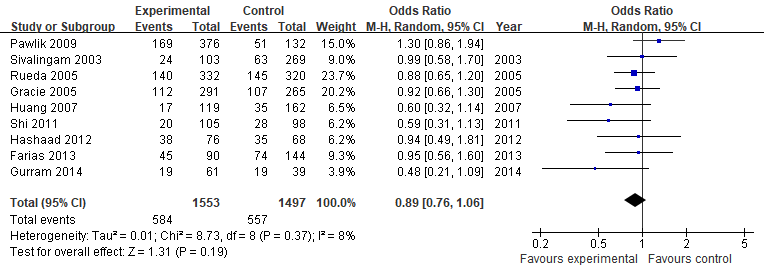


sFig.10 Forest plot of association between IL-18 -137C/G polymorphisms and RA susceptibility in heterozygote model for the overall population
